# Diet and physical activity and metabolic disorders in patients with schizophrenia and bipolar affective disorder in the Polish population

**DOI:** 10.7717/peerj.15617

**Published:** 2023-07-11

**Authors:** Magdalena Głodek, Maria Skibinska, Aleksandra Suwalska

**Affiliations:** 1 Department of Mental Health, Chair of Psychiatry, Poznan University of Medical Sciences, Poznań, Poland; 2 Department of Adult Psychiatry, Chair of Psychiatry, Poznan University of Medical Sciences, Poznań, Poland; 3 Department of Genetics in Psychiatry, Chair of Psychiatry, Poznan University of Medical Sciences, Poznań, Poland

**Keywords:** Schizophrenia, Bipolar disorder, Metabolic disorder, Diet, Physical activity

## Abstract

**Introduction:**

There are numerous reports of a higher prevalence of metabolic disorders in patients with schizophrenia and bipolar disorder (BD), yet its connections to diet and physical activity remain not fully explained. This article aimed to evaluate diet, physical activity and selected biochemical and anthropometric parameters associated with metabolism in patients with schizophrenia and BD and to analyse the relationships between these variables in the subjects.

**Materials and Methods:**

A total of 126 adults participated in the study: 47 patients with schizophrenia, 54 patients with BD and 25 patients in mental illness remission (reference group). Data were collected on the underlying illness and concomitant illnesses, and the severity of symptoms of the current episode was assessed using the following scales: PANSS, MADRS and YMRS. An assessment of the subjects’ diet (KomPAN questionnaire) and their physical activity (International Physical Activity Questionnaire) was carried out. Anthropometric and blood pressure measurements were taken and BMI and WHR were calculated. Serum concentrations of fasting glucose, TSH, total cholesterol, LDL and HDL fractions, triglycerides and leptin, ghrelin and resistin were determined. For statistical analysis, the significance level was set at 0.05. For multiple comparisons one way ANOVA or Kruskal Wallis were used with *post hoc* Tukey and Dunn tests, respectively. To determine correlation of variables, Pearson’s linear correlation coefficient or Spearman’s rank correlation coefficient were used.

**Results:**

A total of 50.8% of the subjects had at least one metabolic disorder—most commonly excessive body weight (66.7%) and abdominal obesity (64.3%). Patients did not differ significantly in terms of physical activity, but they did differ in mean time spent sitting—with this being significantly longer for all groups than in the general population. The subjects differed in diet: patients with BD consumed less unhealthy foods than patients with schizophrenia. The highest correlations between physical activity, diet and variables defining metabolic disorders were found in patients with BD. Only in patients with schizophrenia were there significant correlations between the course of the disease and physical activity.

**Discussion:**

The results suggest the existence of associations between diet, physical activity, and metabolic disorders in both BD and schizophrenia patients. They also suggest a tendency among those patients to spend long periods of time sitting.

## Introduction

The prevalence of metabolic disorders in the population of people with severe mental illness (SMI) has been the subject of research for many years (Ferns, 2018). The prevalence of both isolated metabolic disorders and metabolic syndrome (MS) in patients with SMI is indicated to be significantly higher than in healthy subjects (Penninx & Lange, 2018). According to a large meta-analysis, the risk of MS, defined as the co-occurrence of at least three of the following five criteria: central obesity, high blood pressure, hyperglycaemia, reduced HDL levels, and elevated TG values, is 58% higher in patients with SMI than in the general population (Vancampfort et al., 2015). Furthermore, studies have also described a higher prevalence of MS and its components in patients with first-episode psychosis, not only in those with a long history of schizophrenia (SCHI) (Sahpolat & Ari, 2021). A serious consequence of metabolic disorders is an increased risk of cardiovascular disease, which is the most common cause of premature death in patients with SMI (Penninx & Lange, 2018).

There are multiple factors contributing to the development of MS in patients with BD and SCHI—including smoking, excessive weight, medication, inflammation, adipokine dysregulation, and lifestyle (Mednova et al., 2020; Fourrier, Singhal & Baune, 2019; Heald et al., 2017). Among the postulated reasons for the higher incidence of metabolic disorders in patients with SMI are diet and physical activity (Dipasquale et al., 2013; Vancampfort et al., 2017). A systematic review of the literature on dietary patterns in patients with SCHI indicates the presence of several prominent dietary trends (Dipasquale et al., 2013): among these, a diet low in fibre and characterised by low fruit intake and high in saturated fat is the most frequently mentioned (Henderson et al., 2006), as well as a significantly increased caloric supply and low supply of mono- and polyunsaturated fatty acids compared to the general population (Hasnain et al., 2010). In contrast, data from cross-sectional studies suggest that patients with bipolar affective disorder (BD) follow less healthy dietary patterns than the healthy population (Lopresti & Jacka, 2015). Another study compared the diet of persons with BD in remission and healthy persons in the Polish population—it has been shown that sick persons adhered significantly less to the Mediterranean diet (Łojko, Stelmach-Mardas & Suwalska, 2019). Physical activity of patients with SMI was also studied: the meta-analysis has shown that these patients spend a significant part of the day in a sedentary position, and the time spent per day by patients on moderate and vigorous physical activity was significantly lower compared to healthy subjects (Vancampfort et al., 2017). Apart from often being excessively sedentary, many patients with SCHI and BD tend to prefer low-intensity activities and find themselves being too tired to engage in physical activity, despite knowing the health benefits resulting from regular exercises (Tew et al., 2023). Research is growing on developing interventions aimed towards increasing physical activity in patients with SCHI, however the effects of such interventions tend to be modest (Orleans-Pobee et al., 2022).

Research on diet and physical activity in people with SMI has also investigated the relationships between these variables and the course of mental illness. A systematic review of the data suggests that there is a relationship between patients’ physical activity and cognitive functioning and the severity of negative symptoms in schizophrenia (Rimes et al., 2015). In contrast, in another study, physical activity has been associated with higher quality of life, fewer depressive symptoms and better functioning in patients with BD (Melo et al., 2016). Despite the growing interest in this topic, the relationships between diet, physical activity, and metabolic disorders in patients with BD and schizophrenia remain underexplored and the number of studies involving patients with bipolar disorder is limited.

The aim of the present article was to evaluate and analyse diet and physical activity, as well as biochemical and anthropometric parameters associated with metabolism in patients with schizophrenia and BD, as well as to compare the study groups and to search for relationships between diet, physical activity, and metabolic disorders.

## Materials and Methods

### Subjects

The study group consisted of 126 adults hospitalised in the Department of Adult Psychiatry of the Poznań University of Medical Sciences or treated as outpatients in the Outpatient Clinic of the Department. The inclusion criteria were: age between 18 and 65; a current diagnosis of schizophrenia or BD made by two psychiatrists according to the ICD-10 diagnostic criteria. In the hospitalised group the hospitalisation period prior to the date of inclusion did not exceed 10 days and subjects with BD met the criteria for a present depressive or manic episode. In the reference group the patients met the criteria for remission of the disease: for BD scoring <10 on the MADRS, <12 on the YMRS and for schizophrenia <25 in the eight key items of the PANSS (P1, P2, P3, N1, N4, N6, G5, G9). The diagnosis was initially confirmed by means of clinical interview led by the main researcher and by revising the subject’s medical records. The exclusion criteria were: a coexistence of substance abuse disorder or severe somatic disorders including neoplastic disease, brain tumor or recent history of stroke, acute uncontrolled thyroid disease, renal or hepatic impairment. The study included 47 subjects with SCHI and 54 subjects with BD (including 32 subjects with a depressive episode and 22 subjects with an episode of mania), as well as 25 patients who, at the time of the study, met the criteria for BD or schizophrenia remission (reference group). All subjects agreed to participate in the study by signing a written informed consent. The study was approved by the Bioethics Committee of the Poznan University of Medical Sciences (Resolution No. 855/18).

## Methods

Clinical data on the underlying illness and concomitant illnesses were collected and the severity of symptoms of the current episode of the illness was assessed using the Positive and Negative Syndrome Scale (PANSS) (Kay, Fiszbein & Opler, 1987; Shafer & Dazzi, 2019), Montgomery-Asberg Depression Rating Scale (MADRS) (Montgomery & Åsberg, 1979; Hengartner et al., 2020) and Young Mania Rating Scale (YMRS) (Young et al., 1978; Weiner et al., 2019) scales. An assessment of the subjects’ diet and eating habits was carried out using a validated tool, the KomPAN questionnaire (Jeżewska-Zychowicz et al., 2014), as well as of their physical activity using the International Physical Activity Questionnaire (IPAQ) (Craig et al., 2003). The subjects’ height, weight, waist and hip circumference, blood pressure were measured and BMI and WHR were calculated. Serum concentrations of fasting glucose, TSH, lipid metabolism parameters including total cholesterol (TC), LDL and HDL fractions and triglycerides (TG) and hormones related to metabolism, *i.e*., leptin, ghrelin and resistin were determined (ELISA method).

### Statistical analysis

Calculations were performed using Dell Inc. (2016) Dell Statistica (data analysis software system), version 13. and PQStat Software (2020) PQStat v.1.8.0.388. The statistical significance of the relationships and differences tested was determined at the significance level of α = 0.05. The normality of the distributions of the variables was assessed using the Shapiro-Wilk test. Quantitative variables were presented by mean, standard deviation and median, while other variables were presented by median and mode. Category parameters were described as *n* (%). For quantitative variables with a normal distribution, the study used parametric tests: t-student to compare two mean values with each other, variance analysis (ANOVA) with Tukey’s *post hoc* test to compare multiple groups against a variable and determine in which groups the variables differed significantly, and Pearson’s linear correlation coefficient to check the association of quantitative variables with a linear relationship. For quantitative variables not following a normal distribution, non-parametric tests were used: Mann-Whitney for comparing two groups against a quantitative variable, Kruskal-Wallis with Dunn’s *post hoc* test for comparing multiple groups against a quantitative variable and determining in which groups significant differences were detected, and Spearman’s rank correlation coefficient to determine the correlation of variables. The following tests were used to examine significance for category variables: Chi-square to assess the relationship between the frequency distribution of responses in terms of one variable, Chi-square with Yates correction for expected values lower than five, and Fisher-Freeman-Halton to detect differences in nominal variables between the three groups.

## Results

The subjects did not differ significantly in terms of duration of illness, duration of psychotropic medication and number of relapses. Statistically significant differences existed between the schizophrenia group and the reference group: subjects in the latter group were statistically older (*p* = 0.045) and hospitalised less frequently (*p* = 0.001). Male subjects (*n* = 62) constituted 49.2% of the analysed population. Clinical data characterising the study participants are presented in Table 1. The subjects’ educational level and employment status are presented in Table 2. A listing of medications most commonly taken by the subjects, including psychiatric and non-psychiatric treatment, is presented in Table 3.

**Table 1:** Baseline clinical variables characterising the subjects (mean values, standard deviations, medians) and comparison of variables between groups by diagnostic category (Kruskal-Wallis test).

Variable	Entire group (*n* = 126) F:64, M:62		Patients with schizophrenia (*n* = 47) F:22, M:25		Patients with BD (*n* = 54) F:28, M:26		Reference group (*n* = 25) F:14, M:11		*p* value
	Mean ± SD	Mdn	Mean ± SD	Mdn	Mean ± SD	Mdn	Mean ± SD	Mdn	
Age (years)	42.2 ± 13.1	42	39.1 ± 11.4	38	42.4 ± 13.6	44	47.5 ± 13.6	51	0.045^1^
Duration of illness (years)	12.7 ± 11.1	11	12.2 ± 9	11	12.1 ± 11.1	8	15 ± 9.8	16	0.266
Number of hospitalisations	5.1 ± 4.6	3	6.9 ± 5.1	5	4.9 ± 4.2	3	2.4 ± 2.9	1	0.001^1^
Number of relapses/episodes	7.4 ± 4.9	6	7.4 ± 5.2	6	8.3 ± 4.9	7	5.6 ± 4.1	4	0.054
Psychotropic medication intake period (years)	11.8 ± 9.7	9	11.4 ± 8.3	9.5	11.3 ± 10.7	8	13.7 ± 9.9	14	0.358

**Note:**

1 Significant differences between patients with schizophrenia and the reference group (Dunn’s *post hoc* test).

**Table 2:** Educational level and employment status of the subjects.

Education	Primary education	Basic vocational education		Secondary education		University degree	
N of the subjects	12	10		60		44	
**Employment status**	**Retired/on a disability pension**	**Unemployed/on parental leave**	**Part-time job**		**Permanent employment**		**Still obtaining education**
N of the subjects	46	25	8		42		5

**Table 3:** Types of medication most commonly taken by the subjects.

Medication	Entire group (*n* = 126)	Patients with schizophrenia (*n* = 47)	Patients with BD (*n* = 54)	Reference group (*n* = 25)
Lithium	43	4	23	16
Valproate	24	2	17	5
Typical antipsychotics	10	7	2	1
Atypical antipsychotics	114	42	49	23
Clozapine	25	17	4	4
SSRIs	26	11	9	6
SNRIs	31	2	19	10
Other antidepressants	15	4	8	3
Benzodiazepines	34	18	16	0
Antihypertensive drugs	22	5	8	9
Thyroid hormones	22	5	10	7
Lipid-lowering drugs	8	3	3	2
Anitdiabetic agents	13	5	8	0

The severity of illness symptoms was assessed in the subjects. In the BD group, patients with a depressive episode scored an average of 31 ± 5 points on the MADRS (Mdn = 32), while patients with a manic episode scored an average of 29 ± 6 points on the YMRS (Mdn = 28). The schizophrenia group had a mean PANSS score of 91 ± 11 points (Mdn = 92). Respondents in the reference group met the criteria for remission of the illness, scoring, depending on the diagnosis: <10 points on the MADRS, <12 points on the YMRS or <25 points on the PANSS for the eight key items (P1, P2, P3, N1, N4, N6, G5, G9) of this scale (Zimmerman, Posternak & Chelminski, 2004; Perugi et al., 2018; Andreasen et al., 2005).

### Metabolic disorders

There were no statistically significant differences between the study groups in the occurrence of comorbidities (chi-square test, *p* = 0.148). In 64 patients, at least one chronic disease other than mental illness was found to co-occur—the most common were hypothyroidism (18.3%) and hypertension (13.5%). An analysis of biochemical parameters and anthropometric measurements in the study groups was performed, with the division of BD patients into those with a manic episode and those with a depressive episode. The mean values of the determined parameters are presented in Table 4. No significant differences were found between the groups for the following variables: BMI (*p* = 0.459), WHR (*p* = 0.399), diastolic blood pressure (*p* = 0.354), TSH (*p* = 0.572), glucose (*p* = 0.076), TC (*p* = 0.208), HDL (*p* = 0.086), TG (*p* = 0.554). Statistically significant differences were found for systolic blood pressure (*p* = 0.018) between the reference group and BD patients with a depressive episode and for LDL between the reference group and BD patients with an episode of mania (*p* = 0.047). In both cases, the highest values of the variables were observed in the reference group. It was also determined what proportion of patients in the study groups met the criteria for the presence of or had previously been diagnosed with each of the metabolic disorders. The prevalence of metabolic disorders in the analysed subgroups is presented in Table 5. The majority of the subjects were found to have at least one metabolic disorder. The most common was excessive body weight—overweight defined according to the BMI criterion was present in 66.7% of the subjects, while abdominal obesity defined by the WHR index was present in 64.3% of the subjects. The latter was significantly more frequent in women than in men in the study population (in 82.8% of women and 45.2% of men). Other common metabolic disorders included dyslipidaemia in the form of elevated LDL fraction cholesterol values (49.2%) and hypertriglyceridaemia (43.6%).

**Table 4:** Chosen results of the anthropometric and biochemical measurements in the studied groups (mean values, standard deviations, medians).

Variable	Patients with schizophrenia (*n* = 47)		Patients with BD—depression episode (*n* = 32)		Patients with BD—manic episode (*n* = 22)		Reference group (*n* = 25)	
	Mean ± SD	Mdn	Mean ± SD	Mdn	Mean ± SD	Mdn	Mean ± SD	Mdn
BMI (kg/m^2^)	27.5 ± 5.6	26.6	28.0 ± 6.3	28.5	27.6 ± 6.6	26.1	29.1 ± 4.9	28.6
WHR	0.95 ± 0.08	0.94	0.93 ± 0.11	0.94	0.93 ± 0.10	0.92	0.92 ± 0.06	0.91
Systolic BP (mm Hg)	118 ± 14	120	114 ± 15	115	120 ± 12	120	125 ± 12	130
Diastolic BP (mm Hg)	78 ± 8	80	76 ± 11	78	77 ± 9	76	81 ± 9	82
TSH serum level (µU/ml)	1.50 ± 0.78	1.42	1.78 ± 1.44	1.38	1.48 ± 0.65	1.50	1.98 ± 1.54	1.72
Fasting blood sugar level (mg/dl)	93 ± 14	92	92 ± 16	88	100 ± 44	87	101 ± 13	98
TC serum level (mg/dl)	194 ± 43	195	211 ± 51	207	189 ± 29	192	220 ± 66	204
HDL serum level (mg/dl)	49 ± 15	46	49 ± 11	49	53 ± 18	47	58 ± 14	58
LDL serum level (mg/dl)	114 ± 35	113	128 ± 41	121	101 ± 25	97	135 ± 40	131
TG serum level (mg/dl)	157 ± 86	137	164 ± 95	140	177 ± 97	187	139 ± 65	126

**Table 5:** Prevalence of selected metabolic disorders among the subjects.

Fulfilled metabolic disorder criteria	Patients with schizophrenia (*n* = 47)	Patients with BD—depression episode (*n* = 32)	Patients with BD—manic episode (*n* = 22)	Reference group (*n* = 25)
	*n* affected (group %)	*n* affected (group %)	*n* affected (group %)	*n* affected (group %)
BMI >25 kg/m^2^	30 (63.8%)	20 (62.5%)	14 (63.6%)	20 (80.0%)
BMI >30 kg/m^2^	14 (29.8%)	12 (37.5%)	4 (18.2%)	10 (40.0%)
WHR ≥0.85 (female)	21 (95.4%)	11 (64.7%)	8 (72.3%)	13 (92.9%)
WHR ≥1.0 (male)	11 (44.0%)	8 (53.3%)	5 (45.4%)	4 (36.4%)
Systolic BP ≥140 mm Hg and/or diastolic BP ≥90 mm Hg or previous diagnosis of hypertension	16 (34.0%)	10 (31.2%)	8 (36.4%)	13 (52.0%)
TSH >4.0 µU/ml or previous diagnosis of hypothyroidism	6 (12.8%)	10 (31.2%)	2 (9.1%)	8 (32.0%)
Fasting blood sugar ≥100 mg/dl or previous diagnosis of type 2 diabetes	13 (27.7%)	7 (21.9%)	6 (27.3%)	11 (44.0%)
Serum HDL <45 mg/dl (female) or previous diagnosis of dyslipidemia	6 (12.8%)	4 (23.5%)	4 (36.4%)	0
Serum HDL <40 mg/dl (male) or previous diagnosis of dyslipidemia	11 (23.4%)	4 (26.7%)	3 (27.3%)	1 (9.1%)
Serum LDL ≥115 mg/dl or previous diagnosis of dyslipidemia	21 (44.7%)	19 (59.4%)	8 (36.4%)	14 (56.0%)
Serum TG ≥150 mg/dl	19 (40.4%)	14 (43.7%)	12 (54.5%)	10 (40.0%)

Analysis of the serum levels of leptin, resistin and ghrelin in the patients was performed, showing no statistically significant differences in the values of any of the analysed hormones between the study groups (*p* = 0.647 for resistin, *p* = 0.083 for leptin, *p* = 0.563 for ghrelin).

### Physical activity

An analysis of the patients’ physical activity by group was carried out, taking into account five variables obtained using the IPAQ questionnaire: total energy expenditure related to walking, moderate and vigorous, as well as cumulative physical activity during the week (all expressed in total MET-min/week), and total time spent sitting during the week expressed in minutes—significant differences were found between the study groups. The results obtained are presented in Table 6.

**Table 6:** Physical activity of patients in the study groups and comparison of variables between groups (ANOVA and Tukey’s HSD or Kruskal-Wallis test, respectively).

IPAQ variable	Patients with schizophrenia (*n* = 47)		Patients with BD—depression episode (*n* = 32)		Patients with BD—manic episode (*n* = 22)		Reference group (*n* = 25)		*p* value	F/H, df effect	*Post hoc*	Effect size
	Mean ± SD	Mdn	Mean ± SD	Mdn	Mean ± SD	Mdn	Mean ± SD	Mdn				
Total activity (MET-min/week)	2,388 ± 3,825	1,002	2,404 ± 2,928	1,170	3,914 ± 3,575	2,811	2,565 ± 2,502	1,620	0.052	7.718,3		0.777
Walking (MET-min/week)	718 ± 1,117	297	756 ± 1,154	182	1,601 ± 2,216	866	865 ± 1,028	594	0.062	7.188, 3		0.726
Moderate activity (MET-min/week)	1,079 ± 1,814	360	1,415 ± 2,272	465	2,149 ± 3,191	735	1,590 ± 2,507	720	0.295	3.707, 3		0.283
Vigorous activity (MET-min/week)	591 ± 3,338	0	233 ± 1,030	0	164 ± 3,575	0	109 ± 410	0	0.943	0.384, 3		0.073
Total sitting time/week (min)	4,761 ± 1,178	5,040	4,184 ± 206	4,410	3,440 ± 1,623	3,180	4,066 ± 950	4,140	**0.001** ^1^	5.480, 2	Schizophrenia *vs* manic episode **0.004**^1^	0.932

Variable	ANOVA; the results are significant with *p* < 0.05000							
	SS effect	df effect	MS effect	SS error	df error	MS error	F	*p*
Total sitting time/week	27,670,605	3	9,223,535	205,326,049	122	1,683,000	5,480,412	0.001445

Group	Tukey’s HSD (unequal N); variable total sitting time/week; the results are significant with *p* < 0.05000			
	**{1} M = 4,760.9**	**{2} M = 4,184.1**	**{3} M = 3,440.5**	**{4} M = 4,065.6**
Schizophrenia {1}		0.283720	**0.004111** ^1^	0.230223
BD—depression {2}	0.283720		0.227523	0.988379
BD—mania {3}	**0.004111** ^1^	0.227523		0.379593
Reference group {4}	0.230223	0.988379	0.379593	

**Note:**

1 significant differences between the compared groups.

Statistically significant differences were only observed in the total amount of time the subjects spent sitting per week, while differences in the amount of energy spent per week walking were on the verge of statistical significance. In both cases, the highest values were observed in the BD group during a manic episode. All variables examined were highly scattered, resulting from significant differences in physical activity between patients in each group—some patients exercised regularly, while the activity of others was very low.

Taking into consideration a possible influence of hospitalisation on the subjects’ physical activity routine and thus on the IPAQ score, a comparison between inpatients and outpatients was carried out. No statistically significant difference was found between total physical activity levels (total MET-min/week) of the reference group and hospitalised subjects (Mann-Whitney u-test, *p* = 0.349). Total sitting time per week was also compared between the inpatients and outpatients—again, no statistically significant difference was found between those two groups (t-student test, *p* = 0.463).

The associations between the severity of the symptoms of the current episode of the illness and its course and the physical activity of the patients in each group were also analysed. In the schizophrenia group, positive correlations were found between total time spent sitting and number of hospitalisations (r-Spearman = 0.378, *p* = 0.008), number of relapses (r-Spearman = 0.437, *p* = 0.002) and symptom severity: higher PANSS scores were associated with longer time spent sitting (r-Spearman = 0.404, *p* = 0.005). Higher PANSS scores were also associated with lower total physical activity of the subjects (r-Spearman = −0.307, *p* = 0.036).

### Diet of the subjects

An analysis of the patients’ diets was carried out—the subjects mostly ate three meals a day and often snacked between meals (at least once a day). Only patients in the reference group ate most or all of their meals at fixed times. Patients with schizophrenia most often consumed sweets as a snack, while the other study groups consumed fruit. The study groups were compared in terms of diet quality: for this purpose, two indices were calculated for each patient, according to the procedure for compiling data from the KomPAN questionnaire: the pro-healthy diet index (pHDI-10) and the unhealthy diet index (nHDI-14). A significant difference in nHDI-14 values was found between the schizophrenia and BD groups (t-student test, *p* = 0.015)—patients with schizophrenia scored higher mean nHDI-14 index values than patients with BD (in both mania and depressive episodes). Statistically significant differences were also observed between patients with a manic episode and a depressive episode in the course of BD in terms of mean nHDI-14 values (t-student test, *p* = 0.038)—the first group scored significantly higher on this index. The dietary quality of all subjects in the disease aggravation period and patients in the reference group was also compared (Mann-Whitney u-test). Neither pHDI-10 (*p* = 0.487) nor nHDI-14 (*p* = 0.091) values showed significant differences between the subjects. No association between diet and severity of disease symptoms was detected in either study group.

### Physical activity and metabolic parameters in subjects

Relationships were sought between the subjects’ physical activity and selected biochemical parameters and anthropometric variables related to metabolism. Relationships were examined independently in the diagnostic groups of patients with BD, patients with schizophrenia and the reference group.

Table 7 shows statistically significant correlations between variables defining metabolic disorders and total physical activity in patients with BD. There was also a statistically significant positive correlation between WHR and total time spent sitting by BD patients: the WHR of BD patients increased with the length of time the subjects spent sitting—a doubling of time spent sitting/week was associated with an average increase in WHR of more than 0.45 (r Pearson = 0.300, *p* = 0.031). The relationships described above were not present in the subjects with schizophrenia. In the reference group, however, a strongly positive relationship was present between HDL fraction cholesterol values and total weekly physical activity (R Spearman = 0.526, *p* = 0.007).

**Table 7:** Spearman’s rank order correlations and correlation matrix for metabolism-related variables and total physical activity level (total MET-min/week)—BD patient group.

Correlated variables	*n* subjects		R Spearman correlation coefficient			*p* value
Total MET-min/week and BMI	54		−0.305			0.025^1^
Total MET-min/week and WHR	54		−0.315			0.020^1^
Total MET-min/week and LDL	52		−0.307			0.027^1^
Total MET-min/week and TC	53		−0.318			0.020^1^

Variable	Correlation matrix					
	Total MET-min/week	BMI		WHR	LDL	TC
Total MET-min/week	1,000,000					
BMI	−0.305483	1,000,000				
WHR	−0.314909	0.466245		1,000,000		
LDL	−0.306598	0.214873		−0.000342	1,000,000	
TC	−0.318424	0.218625		0.061252	0.892035	1,000,000

**Note:**

1 statistically significant correlations.

### Diet and metabolic parameters in the subjects

Relationships between the subjects’ diet and selected biochemical parameters and anthropometric variables associated with metabolism were assessed. Positive correlations were found between the unhealthy diet index and blood pressure values in subjects with BD: for systolic BP and nHDI-14 (r Pearson = 0.294, *p* = 0.034), for diastolic BP and nHDI (r Pearson = 0.403, *p* = 0.003). Furthermore, a significant negative relationship was found between WHR and pHDI-10 in BD patients—the higher the health-promoting dietary index values patients achieved, the lower their WHR was (R Spearman = −0.310, *p* = 0.022).

In subjects with schizophrenia, a significant correlation was described between nHDI-14 and HDL fraction cholesterol—high unhealthy diet index values were correlated with low HDL values (R Spearman = −0.322, *p* = 0.027). The reference group showed positive correlations between pHDI-10 and cholesterol values: total cholesterol and pHDI (R Spearman = 0.418, *p* = 0.037) and LDL and pHDI (R Spearman = 0.460, *p* = 0.021).

Significant correlations between frequency of red meat consumption and metabolic parameters were detected in the entire study population. The more red meat the subjects consumed, the higher their WHR (R Spearman = 0.226, *p* = 0.011), systolic (R Spearman = 0.253, *p* = 0.004) and diastolic blood pressure values (R Spearman = 0.275, *p* = 0.002) and the lower their HDL fraction (R Spearman = −0.217, *p* = 0.015). In contrast, the number of meals consumed per day was negatively associated with WHR in the entire study population (R Spearman = −0.180, *p* = 0.043).

**Figure 1:**
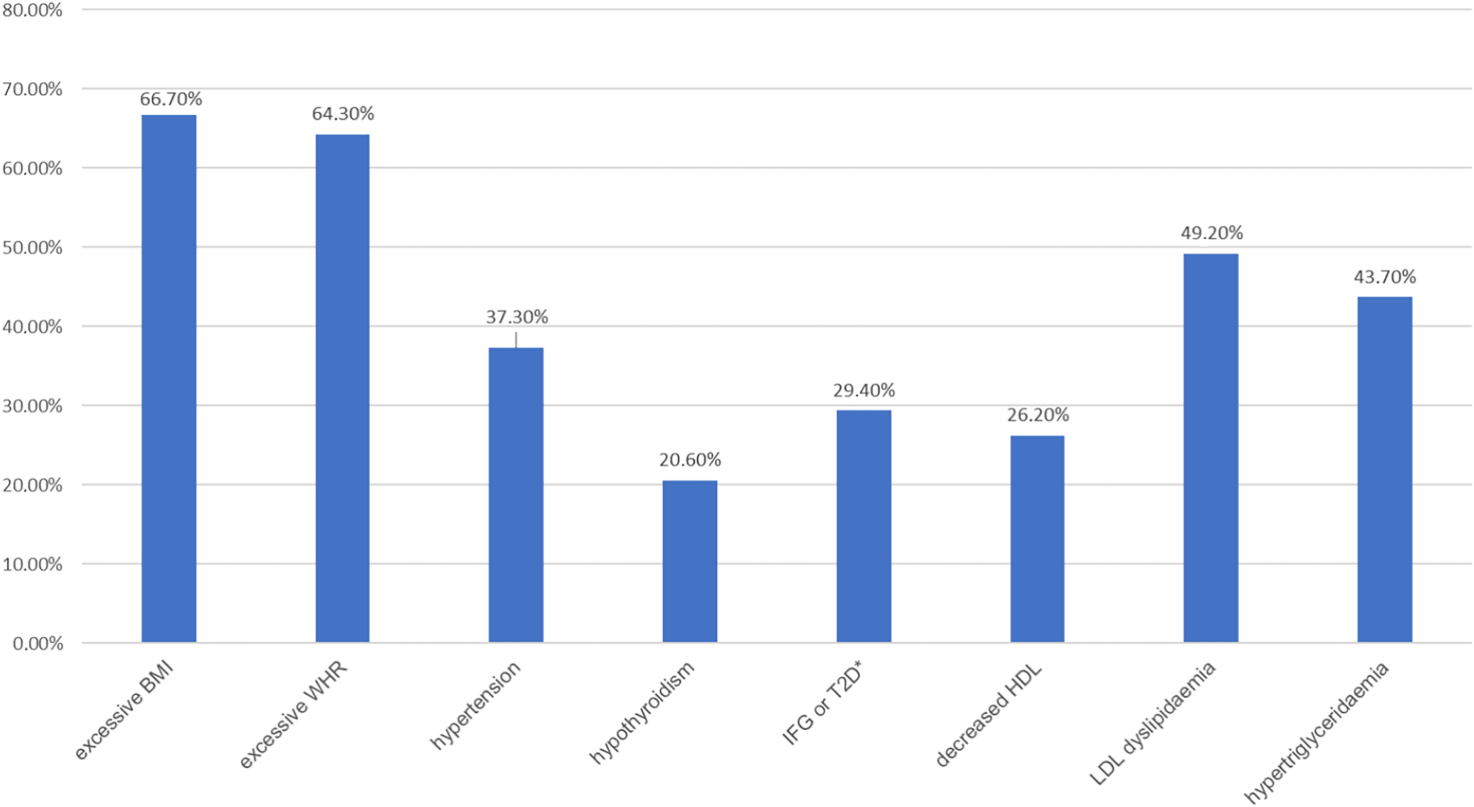
Prevalence of metabolic disorders in the studied population (*n* = 126). An asterisk (*) indicates impaired fasting glucose or type 2 diabetes.

**Figure 2:**
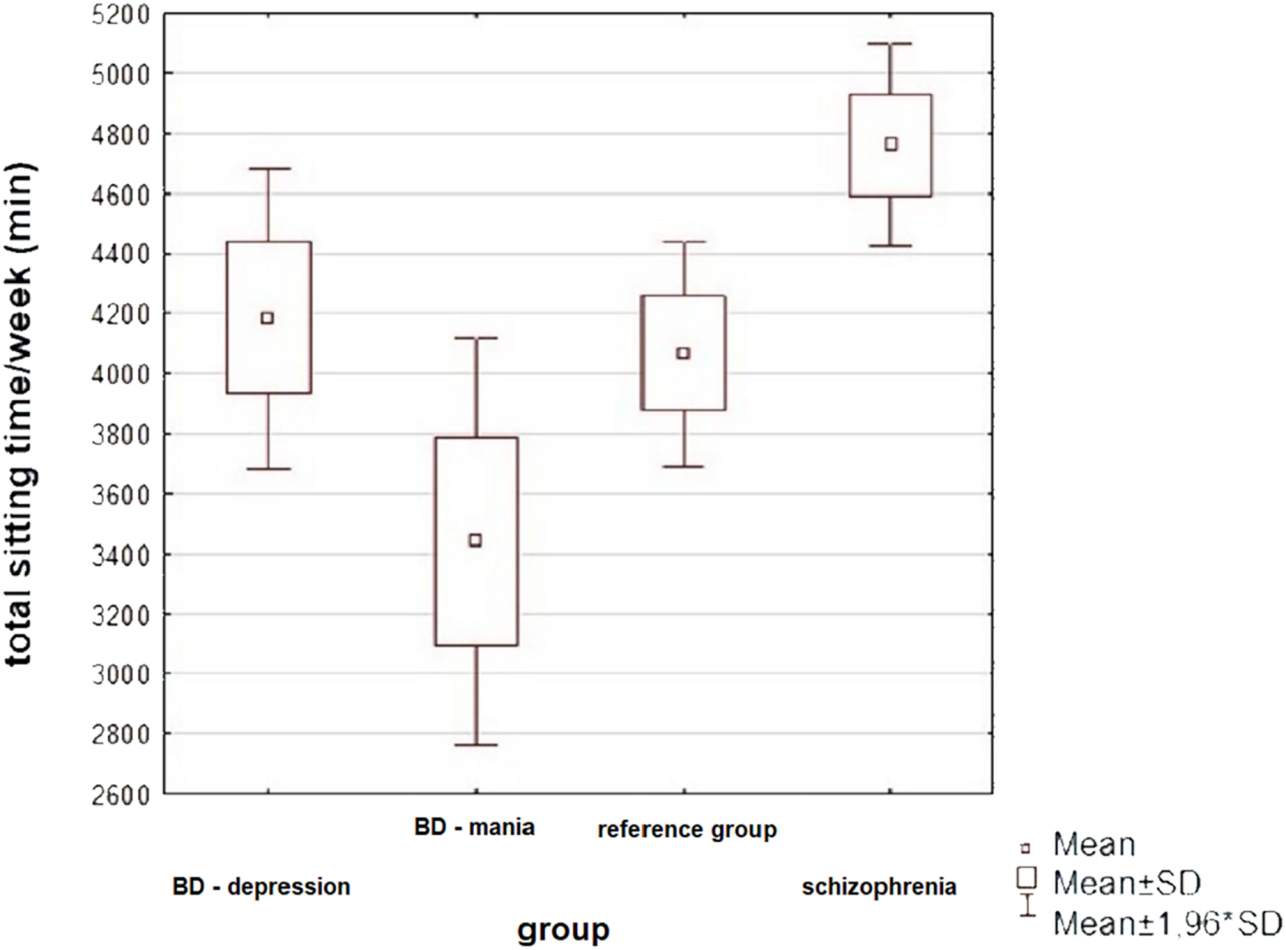
Physical activity of patients in the study groups—a comparison (ANOVA, *p* = 0.001). The subjects differed significantly in total sitting time: patients with SCHI spent the most time sitting, while patients with BD in manic episode—the least (Tukey’s HSD, *p* = 0.004).

**Figure 3:**
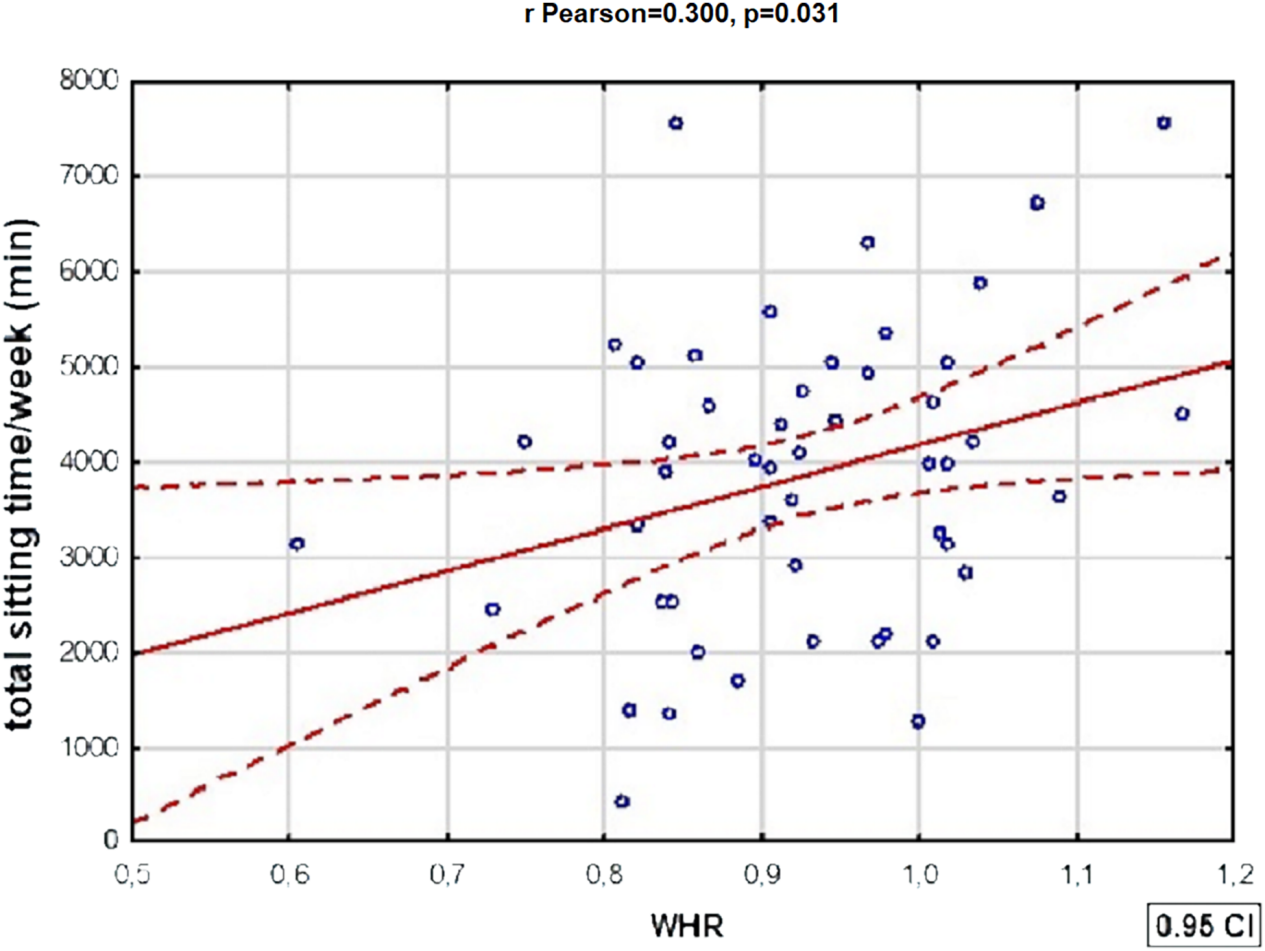
One of the significant correlations between physical activity and variables defining metabolic disorders in patients with BD. There was a statistically significant correlation between WHR and total time spent sitting by BD patients: the WHR of BD patients increased with the length of time spent sitting (r Pearson = 0.300, *p* = 0.031).

**Figure 4:**
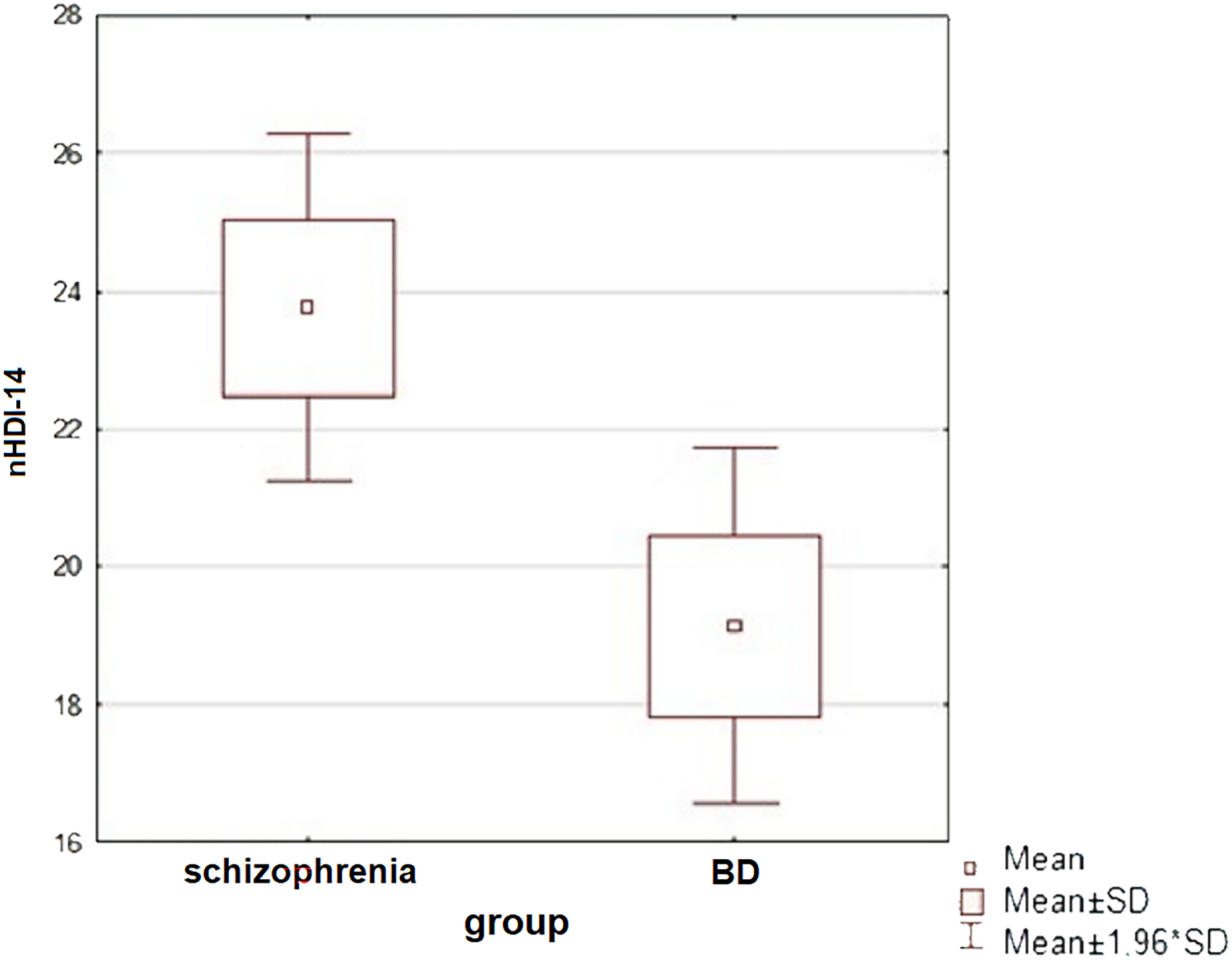
A comparison of the unhealthy diet index values between subjects with schizophrenia and BD (t-student test, *p* = 0.015). A significant difference in nHDI-14 values was found between the schizophrenia and BD groups (t-student test, *p* = 0.015)—patients with schizophrenia consumed more unhealthy products than patients with BD.

**Figure 5:**
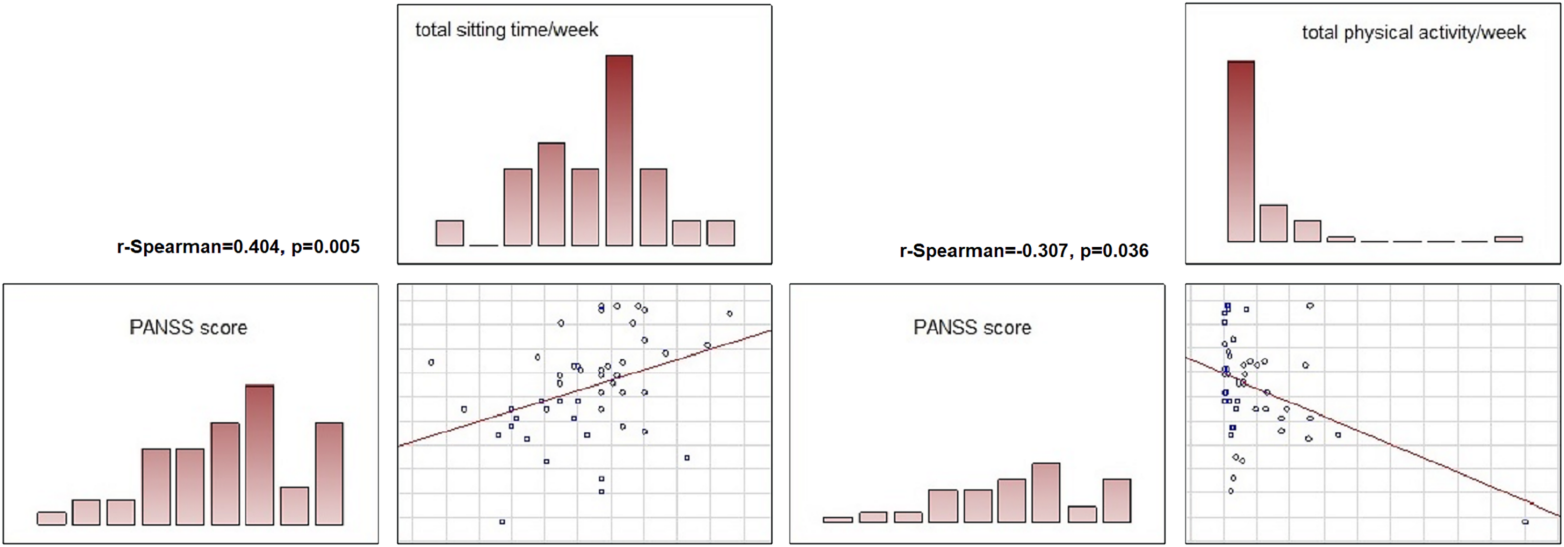
Correlations between symptom severity (PANSS score), total sitting time/week and total physical activity in subjects with schizoprenia. In the schizophrenia group, a significant correlation was found between total time spent sitting and symptom severity: higher PANSS scores were associated with longer time spent sitting (R Spearman = 0.404, *p* = 0.005). Higher PANSS scores were also associated with lower total physical activity of the subjects (R Spearman = −0.307, *p* = 0.036).

A graphic presentation of the most relevant findings of the study is shown in Figs. 1–5.

## Discussion

The subjects did not differ significantly in their level of physical activity irrespective of their primary diagnosis and current episode of illness (for BD). There was a marked tendency for patients experiencing an episode of mania to have a higher total level of physical activity than other patients, but this did not reach the level of statistical significance. The results may indicate that there is no relationship between patients’ perceived increase or decrease in psychomotor drive and actual physical activity performed. Another explanation may also be the phenomenon of behavioural disorganisation resulting from a manic episode, which may prevent patients from systematically performing physical activity at an adequate level of intensity. Vancampfort et al. (2017)’s meta-analysis has shown that patients with BD showed significantly higher levels of physical activity than patients with schizophrenia. In the study presented here, the above trend was also observed, but did not reach the level of statistical significance. Another significant result is the lack of substantial differences in physical activity in patients in the reference group compared to the other study groups, which may suggest that, at least in some patients, achieving remission of the disease does not significantly affect the level of daily physical exertion. In a larger study involving patients with SCHI, physical activity and sedentary behaviour were also not significantly associated with mental, physical and global health composites (Ang, Nurjono & Lee, 2019).

In contrast, the patient groups studied differed significantly in terms of time spent sitting per day. Patients experiencing a manic episode spent on average the least, while patients with schizophrenia spent the most time per day sitting—which is consistent with the results of previous studies describing low activity in patients with schizophrenia (Scheewe et al., 2019). Regardless of the differences observed, all patients were characterised by long time spent sitting per day—the average patient spent 606 min per day in this way. In a meta-analysis including patients with BD, schizophrenia and recurrent depressive disorder, the mean time spent sitting has been lower at 476 min (Vancampfort et al., 2017). Such values were already determined by the authors to be significantly higher than in the healthy population. More recent studies consistently show insufficient physical activity and excessive sedentary behaviour in patients with SCHI and BD (Tew et al., 2023). The result obtained in this study confirms the significant scale of the phenomenon described and indicates the need to take measures to promote a more active lifestyle among patients.

The study groups differed significantly in some aspects of their diet, with unhealthy eating habits more frequently observed in patients with schizophrenia (more frequent consumption of sweets, sweetening of drinks). In relation to the general Polish population, the respondents snacked significantly more frequently between meals: in previous studies, only 17% of Poles have declared eating snacks daily (Feliksiak, 2014), while in the study population it was 57.9% of patients. Respondents consumed vegetables less frequently than the general population—the predominant response was a few times a week compared to “daily” in most Poles (Feliksiak, 2014).

Patients with BD differed from patients with schizophrenia in terms of unhealthy diet index values, with the former group consuming significantly fewer products with harmful effects on health. Similar to the present study, a trend towards poorer dietary intake in patients with schizophrenia compared to those with BD has been described in the literature; such a correlation has also been shown in a systematic review (Teasdale et al., 2019; Bly et al., 2014; Dipasquale et al., 2013). When comparing hospitalised patients with the reference group, the worsening of the illness itself was not shown to be associated with changes in the healthy or poor eating of patients.

Excess body weight was the most common metabolic disorder in the patients participating in the study (66.7%) and did not significantly differentiate the study groups. Relating the data obtained to the prevalence of overweight and obesity in the general population of Poland, it can be concluded that the subjects were only slightly more frequently obese and comparably overweight (Stepaniak et al., 2016). At the same time, the prevalence of abdominal obesity was significantly higher in the study participants than in the general population, where, according to the literature, this disorder was present in 32.2% of men and 45.7% of women (Stepaniak et al., 2016). The observed differences in visceral fat distribution between patients and the general population may, to some extent, explain the greater predisposition of patients with SMI to develop metabolic syndrome and, consequently, cardiovascular disease. Studies have shown that long term changes in body composition and bone mineral density, often resulting from insufficient physical activity, may also contribute to the differences described above (Küçükkubaş & Korkusuz, 2019).

Selected metabolic parameters assessed in this study—namely TSH, BMI, WHR, blood lipid levels and resistin—require weeks or even months to significantly change in values, which could, to some extent, provide a reflection of the participants’ health over a longer period (van der Spoel, Roelfsema & van Heemst, 2021; Acquarone et al., 2019; Takahashi et al., 2010). BMI also correlates with total body fat percentage (Küçükkubaş & Korkusuz, 2020), which constitutes another long term health determinant. Solberg et al. (2016) found that higher serum lipids in patients with SCHI at a 5-year follow-up from baseline were associated with more severe symptoms of the disease and poorer functioning. Another study demonstrated interactive relationships between lipid metabolism and inflammatory markers predicting negative symptom severity in a sample of patients with schizophrenia (Goldsmith et al., 2021). Those findings indicate that serum lipids disbalance may contribute to neuroinflammation, one of the postulated pathologies underlying SMI, including schizophrenia (Mawey et al., 2021; Benedetti et al., 2020). Obesity and overweight (*i.e*., elevated BMI and WHR) have also been clearly linked to neuroinflammation and cognitive decline (Salas-Venegas et al., 2022), which—as described above—could affect two-thirds of the subjects in this study. Thyroid hormones are involved in neuroprotection (Liu & Brent, 2021), hence elevated levels of TSH could also be regarded as a factor increasing the risk of neuronal malfunctioning in the studied population.

It is worth mentioning that even though SCHI and BP share brain metabolism alterations and neuroinflammatory disturbances as pathogenetic factors (Fourrier, Singhal & Baune, 2019; Zuccoli et al., 2017), some differences have also been described. Altamura et al. (2013) described decreased white matter metabolic rates (in large fronto-temporal and cerebellar white matter tracts) in patients with SCHI compared to patients with BD. Another study found that TNFα-related markers in the cortex of people with mood disorders were expressed differently than in people with schizophrenia (Dean et al., 2013). A postmortem transcriptional profiling study found an increased expression of transcripts associated with inflammation across all brain regions examined (prefrontal cortex, striatum, hippocampus) in patients with SCHI, which was not evident in BD or major depressive disorder, or in rat brain following chronic dosing with antipsychotic drugs (Lanz et al., 2019). Those findings may provide one of many explanations for differences in physical activity and diet, as well as metabolic disorders, between patients with SCHI and BD. Interestingly, there are also limited reports on medications affecting human metabolism that might simultaneously reduce neuroinflammation. Data show that some antidiabetic drugs (metformin, thiazolidinediones and compounds targeting the glucagon-like peptide-1 receptor) favorably affect brain metabolism, neuroinflammation, and regeneration (Patrone, Eriksson & Lindholm, 2014). A meta-analysis of randomized clinical trials found that statins administration in apparently healthy people or those with chronic diseases helped reducing biomarkers of low grade inflammation (Milajerdi, Larijani & Esmaillzadeh, 2019). Considering that 54 (42.3%) of all subjects in the present study were taking non-psychiatric medications, the abovementioned effects might also have influenced the prevalence and expression of metabolic disorders in the described group.

In all study participants, there were statistically significant correlations between frequency of red meat consumption and metabolic-defining parameters: WHR, HDL, systolic and diastolic blood pressure. The correlations discussed here are consistent with data for the general population, both in terms of the negative effects of red meat consumption on blood pressure (Schwingshackl et al., 2017) and on the risk of cardiovascular disease (Bronzato & Durante, 2017).

In the present study, statistically significant correlations were observed between physical activity and metabolic parameters in each study group, but most were observed in patients with BD. In all subjects, correlations were observed between selected metabolic-related parameters and total weekly physical activity, whereas total time spent sitting was significantly associated with variables defining metabolic disorders only in the BD group. High physical activity was associated with a lower incidence of general and visceral obesity, as well as dyslipidaemia in BD patients. The results of the study are consistent with previous reports—regular physical exercise and high cardiorespiratory fitness in BD patients were associated with a lower risk of premature mortality (Hearing et al., 2016). Moreover, the positive impact of exercise on mortality rates has recently been described in patients with SMI, not only BD (Baron, Mishrekar & Kazmi, 2022). In addition, in BD patients, longer time spent sitting per week was associated with higher WHR values, whereas no such correlation was observed in the other groups. Evidence from the literature clearly indicates the need to reduce the amount of time patients with SMI spend sitting due to various health benefits, but as yet, it has not been established which behavioural interventions are effective in this regard (Ashdown-Franks et al., 2018). It is also highlighted that dietary and physical activity interventions have high efficacy in improving the metabolic profile of patients with schizophrenia and BD, in contrast to isolated behavioural interventions (Nyboe et al., 2019).

## Conclusions

The results discussed here indicate that both diet and physical activity in patients with schizophrenia and BD are associated with metabolic disorders, independently of other variables such as the pharmacotherapy used. Most pronounced correlations were found between diet, physical activity and BMI and serum levels of lipids. The results also suggest the presence of a tendency to spend a long time sitting among patients with schizophrenia and BD.

The results of this study may provide a baseline for future longitudinal studies to assess the impact of individual clinical variables, such as the severity of the symptoms of the underlying disease, its long-term course, the medications used and other therapeutic approaches, including psychoeducation, on the metabolic health of patients.

### Study limitations

One of the major limitations of the present study is its partially questionnaire-based nature, which raises the issue of objectivisation of the data on diet and physical activity. The study did not include a control group, but a reference group of patients in remission from schizophrenia and bipolar disorder, so comparisons to the general population were made based on data from the Polish literature. In the study population, due to the severe course of mental illness, polypharmacotherapy was often used (including drugs negatively influencing the metabolism such as olanzapine, clozapine, valproate, quetiapine, pregabalin and others), thus no analysis of the correlation between medication intake and parameters associated with metabolism, diet and physical activity was performed. The timeframe of medication use also differed vastly among participants, therefore analyses of a single agent’s influence on the subjects’ metabolic parameters were not conducted. The single-center character of the study also constituted one of its limitations.

## Supplemental Information

10.7717/peerj.15617/supp-1 Supplemental Information 1 Baseline information, scoring and anthropometric and biochemical parameters of every subject.

## Additional Information and Declarations

### Data Availability

The following information was supplied regarding data availability: The raw data is available in the Supplemental File.
